# Epidemiology of ischaemic heart disease in sub-Saharan Africa

**DOI:** 10.5830/CVJA-2012-071

**Published:** 2013-03

**Authors:** Lukwiya Onen Churchill

**Affiliations:** Centre for Chronic Diseases, Gaborone, Botswana

**Keywords:** epidemiology, ischaemic heart disease, sub-Saharan Africa

## Abstract

**Background:**

The epidemiology of ischaemic heart disease (IHD) in sub-Saharan Africa (SSA) remains largely enigmatic. Major obstacles to our understanding of the condition include lack of reliable health statistics, particularly cause-specific mortality data, inadequate diagnostic capabilities, shortage of physicians and cardiologists, and misguided opinions.

**Methods:**

This review of the epidemiology of ischaemic heart disease in sub-Saharan Africa involved a systematic bibliographic MEDLINE search of published data on IHD in SSA over the past century. Search words included epidemiology, ischaemic (coronary) heart disease, myocardial infarction, cardiovascular risk factors and sub-Saharan Africa. Selected data are presented on the prevalence of cardiovascular risk factors and mortality from ischaemic heart disease from different countries representing the main regions of the continent.

**Results:**

Although IHD in SSA remains relatively uncommon, its prevalence is predicted to rise in the next two decades due to the rising prevalence of risk factors, especially hypertension, diabetes, overweight and obesity, physical inactivity, increased tobacco use and dyslipidaemia. It is estimated that age-standardised mortality rates for IHD will rise by 27% in African men and 25% in women by 2015, and by 70 and 74%, respectively by 2030.

**Conclusion:**

Ischaemic heart disease remains relatively uncommon in SSA, despite an increasing prevalence of risk factors, but its incidence is rising. The pace and direction of economic development, rates of urbanisation, and changes in life expectancy resulting from the impact of pre-transitional diseases and violence will be major determinants of the IHD epidemic in SSA. The best window of opportunity for prevention of the emerging epidemic of ischaemic heart disease in sub-Saharan Africa is now.

## Abstract

Over a century ago, Sir Winston Churchill, a renowned British statesman and leader during the Second World War (WWII), made a celebrated visit to Uganda, where he was so moved as to describe it as ‘the Pearl of Africa’. Sir Winston, referring to the quality of intelligence gathered by Western allies during WWII, called Russia a ‘riddle wrapped in a mystery inside an enigma’.

While the same phrase could be used today to describe the epidemiology of ischaemic heart disease (IHD) in sub-Saharan Africa (SSA) because of many puzzles and lingering myths, what is enigmatic is the contempt with which the potential threat of IHD has been treated at various levels of health sectors, governments and international agencies. A recent change in posture by World Health Organisation (WHO) Regional Office for Africa, with greater focus on non-communicable disease (NCD), and the United Nations high-level meeting on NCD prevention and control in New York on 19–20 September 2011 are good indicators of the recognition of the importance of NCDs and the rapidly unfolding epidemiological landscape catalysed by the birth of conjoined twins, infectious diseases and non-communicable diseases.

The 30th anniversary of the Pan-African Society of Cardiology (PASCAR) conference along with the Third All-Africa Conference on Heart Disease, Diabetes and Stroke took place at Munyonyo Speke Resort in Kampala on the shores of Lake Victoria in May 2011. The warmth of the land, the gentle tropical rain showers interspersed with bright sunshine, and above all, the friendliness of Ugandans must have pervaded the hearts of most foreign delegates to the conference.

This review article will focus on some of the obstacles to our understanding of IHD in SSA. A synopsis of cardiovascular risk factors and their role in IHD in SSA, and selected mortality data on IHD from various countries across the continent are presented in this article. A plea for urgent and concerted action to avert the impending epidemic of IHD in SSA is made.

## Obstacles to our understanding of IHD in SSA

Major obstacles to our understanding of IHD in SSA include lack of reliable statistics on health, life expectancy and disease incidence, and the absence of cause-specific mortality data. This is confounded by lack of diagnostic capabilities in most of SSA, emanating from a shortage of physicians, particularly cardiologists, and lack of appropriate investigations, such as resting 12-lead electrocardiographs (ECGs), exercise ECGs, cardiac biomarkers (troponins, CKMB) and cardiac imaging such as echocardiography, coronary angiography, computed tomography (CT) angiography, intravascular ultrasound scans (IVUS) and radionuclide myocardial perfusion studies.

Resting 12-lead ECGs, although generally more widely available and relatively inexpensive, have limited sensitivity and specificity for the diagnosis of acute coronary syndromes. Furthermore, there are high rates of non-specific ST-segment and T-wave changes suggestive of myocardial ischaemia in up to 10% of asymptomatic African men and 20% of women over the age of 40 years.^1^

Physiologically or pharmacologically induced stress tests are helpful to differentiate cardiac from non-cardiac aetiology of chest pain in patients with inducible ischaemia due to obstructive coronary artery disease. The safe performance of provocative stress testing and IVUS requires appropriate professional competence, careful selection of patients and availability of resuscitation equipment in cases of adverse events during testing. Low autopsy rates often coupled with uncertified deaths outside health facilities exacerbate the situation.

This lack of evidence on IHD in SSA is erroneously reinforced by beliefs that IHD affects only the wealthy and elderly, that it arises from freely acquired risks and that its management is expensive, ineffective and of a lower priority than infectious diseases such as HIV/AIDS, tuberculosis, malaria, and a number of neglected tropical diseases. Moreover, there are strong opinions that IHD in SSA affects mainly small Westernised populations and that it is a less serious cause of morbidity and mortality.^2^ Some of these authorities are of the opinion that cardiovascular risk factors in groups of older Africans, including obesity, diabetes and metabolic disorders are virtually non-existent and that IHD is bound to be a less serious threat, as there are very few black populations in the older age category.^2^

Others have expressed disbelief of the potential epidemic of IHD in SSA in the next few decades and contend that resources should be appropriated to the current threats, particularly rheumatic heart disease and cardiomyopathies.^3^ Additional setbacks accrue from lack of appropriate resources and skills to guide and direct epidemiological studies of ischaemic heart disease; crisis management often focused on acute conditions and infectious diseases; and perpetual uncoordinated approaches to health issues that are often reactionary, leading to neglect of NCDs.

The majority of the 57 countries in the world with critical shortages of health workers are in SSA. The total health workforce density in SSA is the lowest in the world with just 2.3 per 1 000 population, compared to 18.9 and 24.8 per 1 000 population in Europe and the Americas, respectively. In fact, SSA has only 4% of the global number of health workers but 25% of the global burden of disease.^4^

Sadly, some of the myths regarding IHD in SSA are fuelled by the notion that the various cardiovascular disease (CVD) risk factors, although prevalent in urban black Africans, appear to exert their influence in a far less noxious manner than is the case in most Western populations. Also that lipid profiles are generally less atherogenic, leading to suggestions of the ‘genetic resistance’ of black Africans to IHD.

The view that IHD is rare in SSA is rooted in old beliefs arising from earlier authors such as Cook^5^ and Donnison,^6^ and needs to be effectively demystified. Firstly, atherothrombotic cardiovascular disease is a global problem that afflicts every community regardless of region, ethnicity or gender. The burden of cardiovascular disease is increasing rapidly in Africa and it is now a public health problem throughout the African region, particularly hypertension, stroke, cardiomyopathies, and not least, ischaemic heart disease. Rheumatic heart disease is still a major concern.

Scarcity of data on IHD and the non-existence of epidemiological surveillance systems for cardiovascular diseases in most of SSA should not be construed to mean rarity of the disorder. INTERHEART, a global case–control study of acute myocardial infarction (AMI) of 28 000 subjects in 52 countries showed that nine risk factors accounted for 90% of populationattributable risk (PAR) in all regions.^7^ These risk factors included hypertension, diabetes, central obesity, dyslipidaemia, physical inactivity, psychological stress, tobacco use, inadequate intake of fruits and vegetables, and inadequate or no alcohol intake.

Although the results of the INTERHEART study have been challenged on account of it being a case–control study rather than a prospective study, the major contributing individual risk factors for acute myocardial infarction are generally consistent across the globe and reminiscent of the conclusions of the original Framingham Heart study several decades ago, as well as its 30-year follow-up study.^8^,^9^ Some have questioned the reliability of information on some of the cardiovascular risk factors used in the INTERHEART study, for example history of hypertension and diabetes mellitus, and have raised concerns about recall bias regarding diet and psychosocial factors in the setting of devastating effects of index acute myocardial infarction on a person’s mental state. In some parts of SSA, haemoglobinopathies such as haemoglobin S or haemoglobin C might contribute to ischaemic heart disease due to vaso-occlussive crises.

Secondly, despite variations in genetic susceptibilities to IHD in different ethnic groups, the common environmental and traditional coronary heart disease risk factors pathogenetically play their roles through a common final pathway in the development of clinical atherosclerotic heart disease in all ethnic groups. Marked regional differences in the impact of CVDs merely reflect a myriad of factors, among them the level of care, quality of health statistics, and differences in stages of socio-economic, nutritional and epidemiological transition between countries, communities and even between individuals.

Thirdly, as societies undergo ‘urbanisation’, risk-factor levels for CVDs including IHD increase. For instance, only about 5% of Africans were urbanised by 1900. At the start of independence in the 1950s, 14.7% of inhabitants of Africa were urban. In 2000, the urbanisation rate had risen to 37.2%, and by 2015 the rate is expected to hit 45.3% with continually high rates of rural–urban migrations across Africa.^10^

## The burden of cardiovascular risk factors in SSA

## Hypertension

Systemic arterial hypertension poses a special challenge in SSA, with immense socio-economic implications because of its high prevalence, especially in urban dwellers. Hypertension is arguably the most powerful cardiovascular risk factor in the African context and has been declared by the African Union as one of the greatest health challenges to the continent other than HIV/AIDS. The problem is compounded by lack of awareness, frequent under-diagnosis, low levels of control and the severity of its complications.^11^-^13^

Despite the dearth of data and marked variation between and within studies, hypertension is estimated to affect 10 to 30% of Africans, virtually one in six people. In West Africa, hypertension affects 30 to 40% of people aged 65 years or older in rural areas, and approximately 50% of semi-urban dwellers. In the mixed population (Coloureds) of South Africa, 50 to 60% of people over the age of 65 years have hypertension. These figures approximate the 60 to 70% prevalence of hypertension in African-Americans over 65 years of age.^14^ An estimated 75 to 80 million Africans, more than twice the global estimate of people with HIV/AIDS, had hypertension in 2000. The number of Africans with hypertension will escalate to 150 million by 2025.^15^

The rising prevalence of hypertension in rural settings is of great concern and probably relates to the rapid ‘urbanisation’ of rural dwellers.^15^,^16^ About 40% of Africans with hypertension are undiagnosed, less than 30% of those who are diagnosed with hypertension are on treatment, and less than 20% of those on treatment have optimal blood pressure control (< 140/< 90 mmHg).^13^,^17^-^21^

## Diabetes mellitus and impaired glucose tolerance

In 2010, an estimated 12.1 million people with diabetes mellitus (4.2% of the global estimate of 285 million) were in sub-Saharan Africa.^22^ The following year, diabetes prevalence rose to 14.7 million (4.02% of the global 366 million). By the year 2030, there will be a 90% projected increase in diabetes prevalence in SSA, bringing the number of Africans with diabetes to 28 million.^23^

Nearly 78% of people with diabetes in sub-Saharan Africa are undiagnosed. Heavily populated countries such as Nigeria have three million diabetics, followed by South Africa with 1.9 million.

Fuelling the diabetes epidemic is a large pool of people with impaired glucose tolerance (IGT), totalling an estimated 26.9 million in 2010, and expected to rise to 47.3 million by 2030. Diabetes is associated with a pro-coagulant state, compounding the commonly accompanying insulin resistance and hyperinsulinaemia, and thus contributing to accelerated atherogenesis.

Although diabetes mellitus and pre-diabetes are important cardiovascular risk factors globally, their roles in populations undergoing rapid epidemiological transition are unclear. Atherosclerotic complications of diabetes are likely determined by the pace and degree of affluence, genetic factors, phenotypic heterogeneity of type 2 diabetes, changes in life expectancy, and burden, duration and contribution of other cardiovascular risk factors such as hypertension, dyslipidaemia and tobacco use. In many parts of SSA, micro-angiopathies are the dominant chronic complications of diabetes,^24^-^30^ unlike in the Western world, where macrovascular complications (MAC) predominate.

## Overweight and obesity

Estimates of the prevalence of overweight and obesity vary widely across SSA, but it is generally higher in females than in males and particularly in southern Africa, Mauritius and Seychelles, compared to the rest of the continent. In East and Central Africa the prevalence of overweight (body mass index from > 25 to < 30 kg/m^2^) in women is two to three times higher than in men (Table 1). In Ghana, males appear to be more overweight than women. However, in much of West Africa, southern Africa and in the islands off the east coast of Africa, the prevalence of overweight in men is approximating that of females. This trend towards parity indicates that overweight is now a widespread continental problem in populations of SSA above the age of 15 years.

**Table 1:** Prevalence Of Overweight And Obesity In Females And Males Aged 15 Years And Older In Selected African Countries By Region, 2011

	*Overweight (BMI > 25 kg/m^2^, < 30 kg/m^2^)*		*Obesity (BMI > 30 kg/m^2^)*	
*Region/country*	*Females (%)*	*Males (%)*	*Females (%)*	*Males (%)*
Eastern Africa				
Uganda	23.9	8.2	1.9	0.1
UR Tanzania	28.7	16.8	3.6	0.8
Central Africa				
DR Congo	15.8	5.7	1.1	0.1
Rwanda	20.7	8.1	1.6	0.1
Western Africa				
Nigeria	36.8	26.0	8.1	3.0
Ghana	32.5	35.6	5.9	4.8
Southern Africa				
Botswana	53.5	41.6	17.7	6.9
South Africa	68.5	41.3	36.8	7.6
Islands				
Mauritius	56.8	44.8	22.3	8.0
Seychelles	73.8	63.8	43.2	21.3

DR Congo = Democratic Republic of Congo, UR Tanzania = United Republic of Tanzania. World Health Organisation: WHO Global Infobase: https://apps.who.int/infobase/Comparisons.aspx (Accessed 28 December 2011). Database updated 20/01/2011. Accessed 28 December 2011.

However obesity still has relatively low prevalence rates throughout SSA, ranging between 1.1 and 43.2% in females and 0.1 and 21.3% in males. Populations of southern Africa and the islands of Mauritius and Seychelles exhibit a greater prevalence of obesity, particularly among the women.

## Physical inactivity

There are scant data on the prevalence of physical inactivity in SSA. A WHO report of national surveys in both urban and rural settings in five African countries (Ethiopia, Republic of Congo, Ghana, South Africa and Zimbabwe) in 2003, involving a total of 14 725 individuals aged 18 to 69 years revealed a mean prevalence of physical inactivity in 19.6% of men and 22.9% of women.^31^

Physical inactivity was defined using the International Physical Activity Questionnaire (IPAQ). IPAQ inactive is defined as not meeting any of the following three criteria: three or more days of vigorous activity of at least 20 minutes per day, accumulating at least 1 500 MET-min per week, OR five or more days of moderate-intensity activity or walking of at least 30 minutes per day, OR five or more days of any combination of walking, moderate-intensity or vigorous-intensity activities, achieving a minimum of at least 600 MET-min per week.

**Fig. 1.**
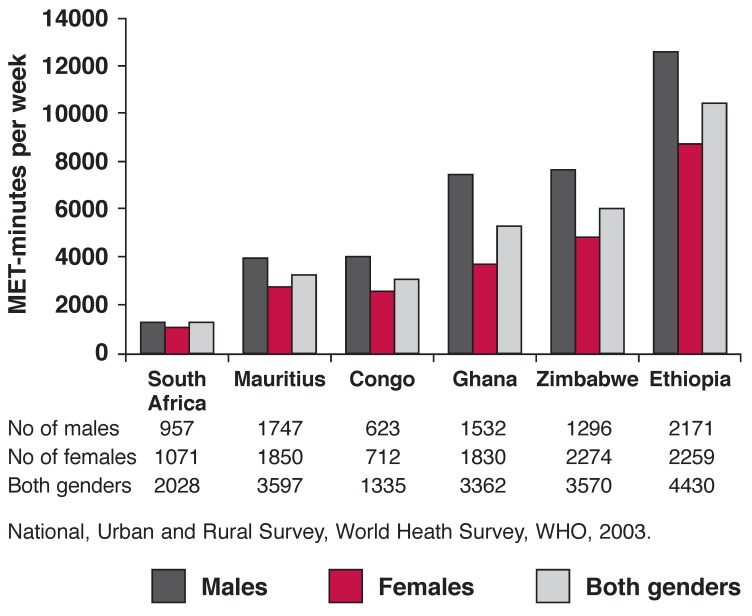
Physical activity in men and women aged 18 to 69 years in selected countries.

Across the continent, low levels of physical activity are reported in women compared to men. According to the WHO survey, a greater number of lazy people are found in southern Africa, Mauritius and Seychelles, while those in the Horn of Africa and in West Africa are relatively more physically active (Table 2, Fig. 1). This observation closely mirrors the reported prevalence of overweight and obesity. There are no consistent national (rural and urban) surveys for similar years or later from other SSA countries.

**Table 2:** Prevalence Of Physical Inactivity In Selected SSA Countries, WHO 2003

*Country*	*Males (%)*	*Females (%)*	*Both genders (%)*
*N/U/R (18–69 years)*	*[95% CI]*	*[95% CI]*	*[95% CI]*
Congo (*n* = 1 335)	23.5	30.2	27.2
M:F = 623:712	[16.5–30.5]	[21.8–38.51]	[20.5–33.9]
Ethiopia (n = 4 430)	9.4	16.0	12.7
M:F = 2 171:2 259	[7.1–11.8]	[13.9–18.2]	[11.0–14.4]
Ghana (n = 3 362)	7.9	15.1	11.5
M:F = 1 532:1 830	[5.9–9.8]	[12.7–17.5]	[9.7–13.3]
South Africa (n = 2 028)	43.0	46.6	44.9
M:F = 957:1071	[37.4–48.6]	[41.4–51.9]	[40.4–49.4]
Zimbabwe (n = 3 570)	14.1	22.0	18.1
M:F = 1 296:2 274	[11.6–16.6]	[19.6–24.5]	[16.4–19.8]

N/U/R = National Urban and Rural Survey. Source: http://infobase.who.int. Accessed 28 December 2011.

The Seychelles Heart study of 2004, reported by Bovet and colleagues in 2007, revealed a disparate prevalence of physical inactivity, ranging from 28 to 58.6% in both genders aged 25 to 64 years, because of variable and subjective operational definitions of physical inactivity using a modification of the WHO STEPS survey questionnaire, which was not identical to the IPAQ.^32^ More surveys are therefore required in many SSA countries using standard questionnaires to provide better insight of the emergence of this cardiovascular risk factor in the continent. There are likely to be wide variations of the levels of physical activities, determined by culture, gender, age, occupation, socio-economic status and levels of education.

## Tobacco use in SSA

Most estimates of tobacco use in SSA vary in their operational definitions. For instance, some surveys have used different age ranges for men and women and between countries. Also, while some surveys considered current tobacco use including smoked and non-smoked tobacco, others have used only daily cigarette smoking. Moreover, these studies were performed in different years, making comparison of prevalence of tobacco use across most African countries problematic.

According to WHO-Afro,^33^ tobacco-smoking rates were considerably lower (< 10%) in countries such as Democratic Republic of Congo, Congo, Ethiopia, Nigeria, Ghana, Swaziland and Lesotho. Countries in Central, West and East Africa had smoking prevalence rates ranging between 10 and 19%. High rates of tobacco use (> 20%) were found mainly in southern Africa, Guinea, Guinea Bissau, Niger, Seychelles and Mauritius. There were no data from certain countries such as Angola, Central African Republic, Gabon and Equatorial Guinea.

It is widely known that some countries on the continent are major tobacco growers. For instance, tobacco accounts for 61 and 23% of export earnings in Malawi and Zimbabwe, respectively. South Africa, Tanzania, Kenya and Nigeria rank closely behind Malawi and Zimbabwe. Continual commercial pressures, price incentives and other subsidies provided by transnational cigarette companies to African farmers, coupled with aggressive marketing and advertisements will drive the prevalence of tobacco use in SSA. It is therefore not surprising that very few African countries have been signatories to the Framework Convention on Tobacco Control Ratification, with countries such as Zimbabwe, Malawi and Eritrea declining to sign the convention altogether.

Table 3 shows age-standardised prevalence estimates for current smokers in males and females aged 25 years or older in 2006 in selected countries. In general, smoking prevalence remains quite low among African women, although increased trends are emerging in young urban women. The prevalence of smoking is 20 to 50 times higher in men than in women across Africa, with estimates of below 2% in women in most SSA countries except in South Africa and Namibia, where smoking prevalence among women was 5.5 and 5.9%, respectively.^34^

**Table 3:** Age-Standardised Prevalence Estimates For Tobacco Smoking (Current Users) In Males And Females Aged 15 Years And Older In Selected Sub-Saharan African Countries By Region, 2006

*Region/country*	*Current smoking prevalence in males aged 15 + years (%)*	*Current smoking prevalence in females aged 15 + years (%)*
Eastern Africa		
Uganda	19.0	2.0
UR Tanzania	24.0	2.0
Central Africa		
DR Congo	13.0	0.6
Malawi	21.0	2.0
Western Africa		
Nigeria	12.0	0.2
Ghana	10.0	0.5
Southern Africa		
Zimbabwe	33.0	2.0
South Africa	29.0	8.0
Islands		
Mauritius	34.0	0.9
Seychelles	32.0	3.0

DR Congo = Democratic Republic of Congo, UR Tanzania = United Republic of Tanzania. Source: https://apps.who.int/infobase/Comparisons.aspx Accessed on 31 December 2011. The figures represent age-standardised prevalence rates, using the standard WHO world population for age, for current tobacco smokers. These figures should be used only to draw comparisons of prevalence between countries and between men and women within a country. These figures are different from the crude data reported in country surveys in Infobase.

Various estimates of smoking prevalence in African men between 1976 and 2005 revealed rates below 10% in many African countries. But in Tanzania, Mozambique, South Africa, Mauritius and Seychelles, smoking prevalence rates ranged between 15 and 30%. Smoking prevalence rates in adults increased substantially across SSA by 2009, especially in Mauritius where a third of adults smoked, closely followed by South Africa, Tanzania, Burkina Faso and Senegal with smoking rates of 27.5, 27.1, 22.0 and 19.8%, respectively. Even in Nigeria and Ghana, where smoking rates were relatively low before 2003, estimated at 6.1% in Nigerian men, 0.1% in Nigerian women, 4.6% in Ghanaian men and 0.2% among Ghanaian women, overall smoking prevalence more than doubled in men to 13 and 10.2% in Nigeria and Ghana, respectively in 2009 but remained quite low in women.

Although deaths from tobacco-related causes probably accounted for only 5 to 7% in African men and 1 to 2% in African women in the year 2000,^34^ by 2030, tobacco is expected to be the greatest contributor of deaths in SSA. Most victims will die 20 to 25 years prematurely of various cancers, respiratory diseases, IHD and other circulatory disorders.

Regrettably, most governments in African countries have avoided action to control smoking for fear of harmful economic consequences on their fragile economies. Without effective tobacco-control measures, SSA risks becoming the biggest global ashtray as many transnational tobacco companies shift their targets to middle- and low-income countries.

## Dyslipidaemia

There is overwhelming epidemiological evidence implicating cholesterol as a cause of atherosclerosis. Most black Africans reportedly have low levels of total cholesterol associated with high high-density lipoprotein (HDL) cholesterol levels.^35^ Higher cholesterol levels however, have been found in diabetic patients from Zimbabwe and Tanzania. The total serum cholesterol was also significantly higher in women than men. Reports from West Africa indicate a worrying trend of dyslipidaemia among patients with either type 1 or type 2 diabetes mellitus.^36^ Data from the Transition of Health during Urbanisation of South Africa (THUSA) study indicate that black South Africans may be protected from IHD because of favourable lipid profiles characterised by low total cholesterol and high HDL cholesterol levels.^37^

In Nigeria, IHD contributes very little to mortality rates in middle-aged men and women, partly because of particularly low mean cholesterol levels.^38^ Different black African communities may be at different stages of their epidemiological transition, as shown in an epidemiological study of coronary heart disease risk factors in the Orange Free State in South Africa.^39^ Table 4 illustrates this point quite vividly. Selected countries representing the different regions of SSA show wide differences in mean total cholesterol levels with a tendency to higher cholesterol levels in females in some countries.

**Table 4:** Estimated Mean Total Cholesterol In Selected African Countries By Region In Females And Males Aged 15 Years And Older, 2011

*Region/country*	*Females mean total cholesterol (mmol/l)*	*Males mean total cholesterol (mmol/l)*
Eastern Africa		
Uganda	4.4	4.7
UR Tanzania	5.2	4.4
Central Africa		
DR Congo	4.3	4.3
Rwanda	4.3	4.3
Western Africa		
Nigeria	3.7	3.6
Ghana	5.9	4.4
Southern Africa		
Botswana	4.7	4.7
South Africa	4.4	4.4
Islands		
Mauritius	5.2	5.2
Seychelles	5.9	5.8

DR Congo = Democratic Republic of Congo, UR Tanzania = United Republic of Tanzania. Source: https://apps.who.int/infobase/Comparisons.aspx Accessed on 31 December 2011.

## The cardiovascular impact of HIV/AIDS

SSA bears a disproportionate share of the global HIV burden. The interaction between HIV infection, acquired immunodeficiency syndrome (AIDS), its treatment with highly active antiretroviral drugs (HAART), and cardiovascular disorders is complex and incompletely understood.

The transformation of HIV/AIDS into a chronic disorder with the advent of antiretroviral drugs is associated with the emergence of certain characteristic cardiovascular risk factors, and raises apprehension about the potential increase in prevalence of cardiovascular diseases, including IHD, in SSA. In Botswana, for instance, where antiretroviral therapy coverage exceeds 90%, AIDS-related deaths declined by approximately 50% between 2002 and 2009.^40^

The repertoire of immunological responses associated with acute and chronic HIV infection is quite complex and will be only highlighted here. Perturbations of cytokine expression, cellular dysfunctions, redistribution of lymphocyte sub-populations, increased cellular turnover and apoptosis are some of the features of general activation of the host’s immune system that characterise chronic HIV infection.^41^ Chronic HIV infection, and not its pharmacological treatment, induces changes in markers of endothelial function.^42^ Untreated HIV infection is also associated with impaired elasticity of both large and small arteries.^43^

Some authors have suggested that HIV infection accelerates atherosclerosis via a pro-inflammatory effect on the endothelial cells through the effects of various cytokines, especially interleukin-6 and D-dimers.^44^,^45^ Other mechanisms of arteriopathy include the direct toxic effects of HIV-associated g1p20 and tat proteins on vascular or cardiac cells. There is also evidence of a hypercoagulable state, which inversely correlates with CD_4_ count.^46^

Although traditional risk factors for cardiovascular diseases might overshadow the role of non-traditional risk factors, there is increasing evidence that young, asymptomatic, HIV-infected men with long-standing HIV disease demonstrate an increased prevalence and degree of coronary atherosclerosis compared with non-HIV-infected patients.^47^ Furthermore, HIV-infected patients tend to develop perturbations in lipid metabolism, characterised by decreased HDL cholesterol and low-density lipoprotein (LDL) cholesterol levels, followed by an increase in plasma triglyceride levels pre-HAART and prior to developing AIDS.^48^

Both traditional and non-traditional risk factors therefore appear to contribute to atherosclerotic disease in HIV-infected patients. Those on HAART, particularly protease inhibitors, develop a myriad of class- and non-class-specific metabolic effects on lipid profiles, glucose levels, insulin sensitivity and anthropometric body changes characteristic of lipodystrophy. Untreated HIV infection may also have a paradoxical overall effect on cardiovascular disease and thereby reduce the risk of ischaemic heart disease because of severe and progressive weight loss, wasting syndrome, hypotension resulting from chronic gastroenteritis, hypoadrenalism and shortened life expectancy associated with advanced AIDS.

**Fig. 2.**
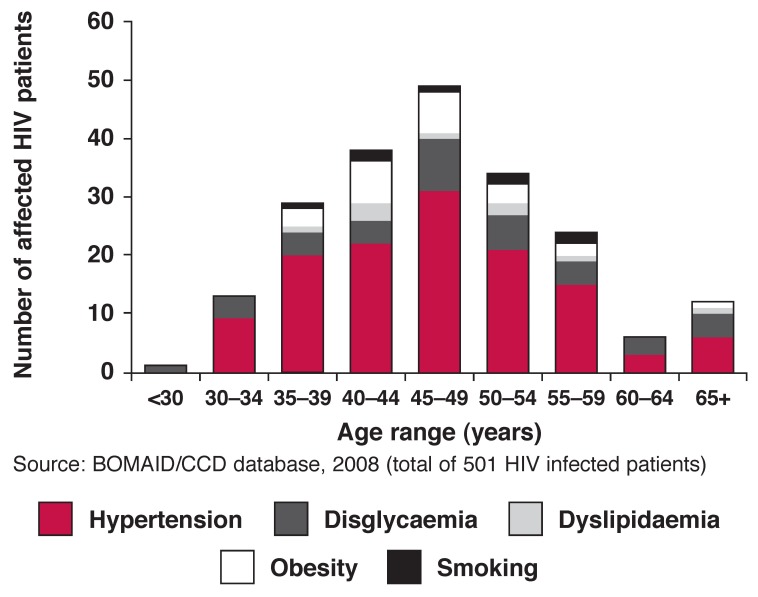
Cardiovascular disease risk factors in HIV-infected patients in Botswana.

Despite the scarcity of data from SSA, there are some indications of overall excess CVD risk factors in HIV-infected patients. Situation analysis in 2008 of 501 HIV-infected patients from Botswana using the database of the Botswana Medical Aid Scheme combined with data from the Centre for Chronic Diseases revealed impressive clustering of hypertension, dyslipidaemia, obesity, dysglycaemia and smoking (Fig. 2). The peak age range for the occurrence of CVD risk factors was about a decade after the peak age for HIV infection in Botswana.

Given the difficulty of determining whether the observed increase in CVD risks were due to HIV itself, treatment with HAART or merely a factor of improved longevity, it would be ideal to perform case–control studies on the prevalence of CVD risk factors and the prevalence of arteriosclerotic cardiovascular endpoints such as IHD, stroke, and peripheral arterial disease in HIV-infected versus age- and gender-matched non-HIV-infected individuals. Also, a comparison of pre-HAART and on-HAART HIV-infected patients would shed light on this grey area. It is important to remember that the enormous impact of HIV/AIDS does not appear to have diminished the impact of chronic cardiovascular diseases on mortality in SSA.^49^

## Reports on IHD in SSA

There are a few scattered reports of IHD in SSA. Kengne and colleagues^50^ collated a total of 356 cases of SSA patients with coronary heart disease (CHD) from four selected countries (Ghana, Cameroon, Senegal and Kenya). They reported a high prevalence of CHD risk factors, which was not surprising in this selected population of patients with established CHD. Males outnumbered females by ratios ranging from 1.3:1 to 6:1, with hypertension in up to two-thirds of the patients. The report highlighted the fact that IHD was by no means rare in these African populations.

The African arm of the INTERHEART study showed that dyslipidaemia, abdominal obesity and tobacco use accounted for greater population-attributable risk in the overall African population, whereas hypertension and diabetes were less prominent risk factors.^51^ However, in black Africans, dyslipidaemia was followed by hypertension, abdominal obesity, diabetes and then tobacco use.

The INTERHEART African study cast doubt on the notion of protective lipid profiles in blacks, as one reason for implicitly low IHD prevalence in Africa. High HDL cholesterol levels in black Africans might be dysfunctional and less protective than generally believed. However, the findings of the INTERHEART African study were at slight variance with reports by Ezzati and colleagues who showed that hypertension, low intake of fruits and vegetables and physical inactivity accounted for population-attributable fractions for ischaemic heart disease mortality of 43, 25 and 20%, respectively, in the Africa region. These were all above the population-attributable fraction of 15% for high cholesterol.^52^

Limitations in diagnostic evaluation of patients with possible IHD might explain, at least in part, the apparent rarity of IHD in SSA. This is illustrated by the study on black South Africans by Joubert and colleagues using data from the Medical University of South Africa (MEDUNSA) stroke data bank. The study showed increased prevalence of CHD with improved diagnostic tools.^53^

History of angina pectoris or myocardial infarction using the Rose questionnaire yielded a prevalence of only 0.7% in 741 black patients with stroke, 71% of whom had cerebral infarction. Resting 12-lead electrocardiography was analysed for the presence of poor R-wave progression in the precordial leads, the presence of pathological Q waves and ST–T wave changes using the Minnesota code in 555 stroke patients, 72% of whom had cerebral infarctions confirmed on computed tomography. Ninety-three of the 555 patients (16.8%) had evidence of coronary artery disease, of whom 81 had features of myocardial ischaemia, eight had pathological Q waves and four patients had features of acute myocardial infarction. There has been longstanding controversy regarding ECG diagnosis of myocardial ischaemia in black Africans.^53^-^57^

Ignoring ECG features of ‘ischaemia’ and ascribing such changes to ‘normal variation’ poses the potential danger of under-diagnosis or misdiagnosis of myocardial ischaemia in black Africans. Rather, future work should attempt to unravel the genetic mechanisms behind abnormal ECG patterns in black Africans.

The combination of clinical assessment, chest radiograph, resting electrocardiography, transthoracic echocardiography and MUGA scanning showed features of CHD in 18 patients (17.6%) in the MEDUNSA study. Scintigraphy with or without dipyridamole infusion in 60 stroke patients in this study revealed features of coronary heart disease in 45% of the patients. Macroscopic and microscopic pathological examinations of the heart and coronary arteries for evidence of infarction in 23 stroke patients in the study revealed the highest rate of myocardial infarction (17.4%).

Observed differential mortality rates in different ethnic groups in multiracial African communities such as South Africans have been at least partly ascribed to different stages of the epidemiological transition. For instance, Norman and colleagues^58^ found that black Africans had approximately 60, 70 and 82% less CHD mortality rates compared to South African Coloureds, whites and Asians, respectively.

Part of the reason for relatively high IHD mortality rates in South African Asians is due to their high prevalence of diabetes mellitus.^59^-^61^ By contrast, mortality from stroke in black Africans exceeds the rates for Coloureds, whites and Asians by 2, 96 and 19%, respectively. However, mortality from hypertensive heart disease in black South Africans was 2.5, nine and three times higher than rates in Coloureds, whites and Asians, respectively.

Bradshaw and colleagues^62^ demonstrated that IHD was the leading cause of death among 71 641 South African men over 60 years, while it was the second most common cause of death among the top causes of deaths in 73 474 women in the year 2000 (Table 5). In South African men aged 15 to 45 years in the same study, IHD was ninth among the top 10 causes of death (1.1%), although it did not feature among the top 10 causes of death in women. HIV/AIDS was the predominant cause of mortality in younger age groups, accounting for 40.7% of deaths in men and 64.4% in women.

**Table 5:** Causes Of Mortality In South African Men And Women > 60 Years In 2000

*Cause of death*	*Percentage (%) in males aged > 60 years [n = 71 641]*	*Cause of death*	*Percentage (%) in females aged > 60 years [n = 73 474]*
Ischaemic heart disease	17.2	Stroke	17.7
Stroke	12.2	Ischaemic heart disease	16.0
COPD	8.0	Hypertensive heart disease	9.8
Tuberculosis	6.4	Diabetes mellitus	7.3
Lower respiratory tract infection	5.1	Lower respiratory tract infection	5.3
Hypertensive heart disease	4.2	COPD	4.4
Cancer of airways	4.1	Nephritis	2.8
Diabetes mellitus	4.0	Tuberculosis	2.7
Cancer of prostate	3.1	Asthma	2.4
Cancer of oesophagus	2.8	Cancer of the breast	1.9

COPD = chronic obstructive pulmonary disease.

In 2005, the WHO estimated 188 000 and 173 000 deaths from IHD in men and women, respectively in SSA.^63^ These age-standardised mortality rates (ASMR) will rise by 27 and 25% in men and women, respectively by the year 2015, and by 70 and 74%, respectively by the year 2030.

Table 6 represents ASMR from IHD in selected countries from the main regions of SSA. Despite higher ASMR in men in mainland Africa, rates in females were close to those in men (Table 6). In Seychelles, ASMR in men was three-fold higher than rates in women, while Mauritius shows the highest ASMR for IHD in both genders, with a male preponderance.

**Table 6:** Age-Standardised Mortality Rates For Ischaemic Heart Disease In The WHO Africa Region, By Selected Countries And Gender, 2002

		*Age-standardised mortality rates for IHD (per 100 000)*	
*Region/country*	*Estimated population (millions)*	*Males*	*Females*
Eastern Africa			
Uganda	25.00	150	120
Tanzania	36.28	147	128
Ethiopia	6.90	149	127
Central Africa			
DR Congo	51.20	166	132
Rwanda	8.27	149	122
Malawi	11.87	152	125
Southern Africa			
Botswana	1.77	142	102
South Africa	44.76	159	99
Mozambique	18.54	124	107
Western Africa			
Nigeria	120.91	160	127
Ghana	20.47	143	114
Cameroon	15.73	154	124
Islands			
Mauritius	1.21	277	161
Seychelles	0.80	151	49

DR Congo = Democratic Republic of Congo, UR Tanzania = United Republic of Tanzania.

Some caveats against current and future projections of mortality data for IHD in SSA include the use of approximations that often embrace substantial uncertainties, especially in the estimation of cause-specific deaths. This huge degree of uncertainty has been attributed to a meagre database on IHD as a specific cause of death in Africa and to the overall low coverage of vital registration.

Despite the heavy toll inflicted by HIV/AIDS in SSA, comparative ASMR across the continent indicate that mortality from IHD matches and already exceeds those from HIV/AIDS in some regions of SSA,^48^,^64^-^65^ except in southern Africa, the epicentre of the HIV/AIDS epidemic (Table 7). In Botswana and South Africa, respectively, there were nine- and three-fold more deaths from HIV/AIDS compared to deaths from IHD. In Mauritius, ASMR for IHD was 274-fold higher than rates from HIV/AIDS, and in Seychelles, the difference was 36-fold. In Ghana, ASMR for IHD was 1.5 times that of HIV/AIDS between 2002 and 2004.

**Table 7:** Comparison Of Age-Standardised Mortality Rates For Ischaemic Heart Disease And Hiv/Aids In The WHO Africa Region In Selected Countries In 2002

		*ASMR (per 100 000)*		*ASMR*
*Region/country*	*Estimated population (millions)*	*IHD*	*HIV/AIDS*	*HIV/AIDS: IHD ratio*
Eastern Africa				
Uganda	25.00	270	555.6	2.06
UR Tanzania	36.28	275	593.2	2.16
Central Africa				
DR Congo	51.20	298	277.7	0.93
Malawi	8.27	271	345.4	1.27
Western Africa				
Nigeria	120.91	287	316.8	1.10
Ghana	20.47	257	174.6	0.66
Southern Africa				
Botswana	1.77	244	2,243.1	9.19
South Africa	44.76	258	840.3	3.26
Islands				
Mauritius	1.21	438	1.6	0.004
Seychelles	0.80	200	5.5	0.03

DR Congo = Democratic Republic of Congo, UR Tanzania = United Republic of Tanzania, ASMR = age-standardised mortality rates. Sources: WHO Global InfoBase http://infobase.who.int; WHO Statistical Information System http://www.who.int/whosis ; Mackay J, Mensah GA. *The Atlas of Heart Disease and Stroke*. Geneva: World Health Organization. 2004. http://www.who.int/cardiovascular_diseases/resources/atlas/en.

## Conclusion

Nearly 40 years ago, Bradlow and colleagues^66^ stated that Africa provided a vast natural laboratory for the study of the aetiology and epidemiology of heart disease. Little appears to have changed in terms of the epidemiology of IHD in SSA. The scarcity of cause-specific data makes a mockery of the case for gitating for greater action plans to combat IHD in SSA amidst a storm of infectious diseases such as HIV/AIDS, tuberculosis and malaria.

We need epidemiological data to make IHD less tentative and unconvincing to sceptics, healthcare providers and policy makers. An important starting point is the establishment of cardiac registries in multiple centres across the continent.

Various tertiary centres of excellence already exist in parts of sub-Saharan Africa for care of acute coronary syndromes and cardiac rehabilitations. However, these facilities are few and far between and are not within the reach or affordability of all of those who need them. As with HIV/AIDS, the fight against the pandemic of cardiovascular diseases must concentrate on primary prevention. Novel approaches must be developed that effectively connect community resources with organised healthcare systems and must integrate both behavioural and biomedical approaches.

IHD remains relatively uncommon in SSA despite an increasing prevalence of risk factors but its incidence is rising. The pace and direction of economic development, rates of urbanisation and changes in life expectancy resulting from the impact of pre-transitional diseases and violence will be major determinants of the IHD epidemic in SSA. The best window of opportunity for concerted action to tackle the emerging epidemic of IHD in SSA is currently shrouded by the lingering burden of infectious diseases.
